# Technical Report of the Use of a Novel Eye Tracking System to Measure Impairment Associated with Mild Traumatic Brain Injury

**DOI:** 10.7759/cureus.1251

**Published:** 2017-05-15

**Authors:** Michael Kelly

**Affiliations:** 1 Procare Medical Associates LLC, Sports Medicine

**Keywords:** concussion, mtbi, eye tracking, ocular, smooth pursuit, dynamic visuomotor synchronization, impairment of attention, predictive timing, eyeguide focus, baseline testing

## Abstract

This technical report details the results of an uncontrolled study of EyeGuide Focus, a 10-second concussion management tool which relies on eye tracking to determine the potential impairment of visual attention, an indicator often of mild traumatic brain injury (mTBI). Essentially, people who can visually keep steady and accurate attention on a moving object in their environment likely suffer from no impairment. However, if after a potential mTBI event, subjects cannot keep attention on a moving object in a normal way as demonstrated on their previous healthy baseline tests. This may indicate possible neurological impairment. Now deployed at multiple locations across the United States, Focus (EyeGuide, Lubbock, Texas, United States) to date, has recorded more than 4,000 test scores. Our data analysis of these results shows the promise of Focus as a low-cost, ocular-based impairment test for assessing potential neurological impairment caused by mTBI in subjects ages eight and older.

## Introduction

The concussion is the most common mild traumatic brain injury (mTBI) seen in contact sports, usually resulting from minor trauma to the head [1-2]. It is a transient neurologic deficit often associated with impairment of consciousness. Minor symptoms can include a headache, dizziness, light sensitivity, abnormal gait, nausea or vomiting. The abnormality may not be observable in the routine neurological examination; even standard neuroimaging diagnostics, such as computerized tomography (CT) and magnetic resonance imaging (MRI) may reveal no abnormality. Therefore, diagnosis is often based on the appearance and severity of the aforementioned minor symptoms.

In sports-related concussion, diagnosis is even more challenging due to factors such as underreporting, underrecognition, and unwitnessed events combined with various psychosocial issues, e.g. peer pressure, apprehension over mandatory game rest or even economic concerns like potential salary loss or educational scholarship revocation. Repeated concussive events without allowing adequate ‘brain rest’ is the most common cause of persistent symptoms lasting more than three months, commonly termed post-concussion syndrome. Effects may be long-term and debilitating, including poor school performance and inability to maintain normal physical activity.

Maruta [3] takes up the challenge of operationalizing brain injury, successfully correlating mTBI with impaired performance on a smooth pursuit eye test. It was previously shown that strength of visual attention is associated with the same areas of the brain that are damaged in concussion [4-5]. Maruta's research shows that predictive timing and essential element of attention is very often impaired in individuals with mTBI.

Predictive timing termed as dynamic visuomotor synchronization (DVS) by Maruta involves constant sensory processing and motor execution of goal-oriented behavior. In 13 concussed and 127 normal subjects between the ages of 18 to 55 years, DVS scores of subjects with mild head injury were worse than 95% of those without the concussion. Also, longitudinal monitoring of injured subjects revealed that their DVS scores improved toward the normal range as they healed. The scores were reproducible with little learning effect.

In detail, the test involved, asking subjects to follow a target stimulus moving clockwise in a circular trajectory with a 10° radius at 0.4 hertz (Hz). DVS was characterized by the variability of the instantaneous gaze positional error in the direction parallel to the target movement. In other words, more variability between the velocity of the target and the subject's eye movement resulted in a higher (worse) DVS score.

Essentially, people who can visually keep steady and accurate attention on a moving object in their environment, what Maruta, et al. characterize as DVS, likely suffer from no impairment. But if people with acceptable visual acuity cannot keep attention on a moving object in a normal way, then the failure to do so may indicate possible neurological impairment.

Building on Maruta's discovery and DVS concept, EyeGuide, an eye tracking hardware and software company located in Lubbock, Texas, United States created the Focus test in order to detect brain injury in youth sports athletes.

The focus is a 10-second test for impaired brain function. Focus can be used immediately after the injury including that on the sidelines during competition. A test subject looks at a small white circle moving in a figure-eight pattern against a black background on a tablet screen. The EyeGuide eye tracking headset records their eye movements during the test. Deviation from expected gaze position during the test (in partial pixels, at 60 Hz) is totaled to yield a test score (the lower is better).

Before the season begins, the athlete takes one test, called the baseline. Then, if an injury is suspected, he or she takes the same test again. The system compares the athlete's score with his or her baseline score as well as the scores of thousands of other, similar athletes in the system. If the score is abnormally high, it would indicate a drop in neurocognitive ability associated with brain injury.

The value of such a test, if effective, would mean fast, low-cost mTBI assessment, especially in sports athletes and could be made available as another reliable tool for athletic trainers and other healthcare professionals tasked with recognizing and managing concussions.

This technical report details the results of an uncontrolled study of EyeGuide Focus which attempted to determine the efficacy of Focus as an impairment measurement instrument using the same DVS analytical method employed by Maruta.

## Technical report

The Focus hardware (EyeGuide, Lubbock, Texas, United States) consists of a head-mounted eye tracking device and a standard 9.7-inch iPad. The iPad runs EyeGuide's proprietary Focus app, which provides roster management and neurological impairment testing capabilities. The eye tracking hardware communicates with the software running on the iPad over Wi-Fi.

In the EyeGuide Focus test, the stimulus (a white filled circle against a black background) starts at the center of the tablet screen and moves clockwise through one cycle of a "lazy 8" path. In other words, starting from the center of the display, the stimulus follows the path of a circle, clockwise around an invisible point in the center-right of the display. When the stimulus reaches the center of the display again, the path changes to a counterclockwise circle around an invisible point in the center-left of the display. Finally, when the stimulus returns to the center of the display again, it begins again to track the right circular path. The test ends after exactly 10 seconds after the stimulus has begun again to track the right circular path.

When the individual's Focus score is computed, data from the first and final seconds of the test are discarded to minimize primacy effects. The stimulus moves quickly enough to traverse the entire "lazy 8" track with some time left over in order to facilitate this sanitation of the input eye velocity data. The stimulus moves at the same velocity during every test.

At the conclusion of the test, the test administrator receives confirmation of a successful or unsuccessful test. If the test is unsuccessful because of technical reasons (such as poor headset fit, athlete head movement during testing), the athlete is re-tested. If the test is successful, the athlete is excused and the athlete’s normative, individual baseline score is recorded for future comparison against potentially impaired scores occurring after concussive-related injury.

Each test including the normative, individual baseline test is assigned a score. To determine the score, the input data points (pupil center coordinates, 60 per second) are first transformed to negate any effect of the athlete having observed the stimulus at an oblique angle. In other words, the points at the extremes of the test are used as guide points to transform all of the points to the coordinate system of the original stimulus on the tablet display screen. This post-test coordinate transformation also eliminates the need for a discrete pre-test calibration step, which further reduces the time taken to complete the test and prevents possible “gaming” of the test by athletes who might purposely perform poorly during calibration.

Then, the final test score is taken to be the sum of the distances between the transformed pupil center coordinates and the actual on-screen stimulus coordinates for the entire duration of the test, that saved the first and last seconds. Thus, a score of zero would indicate flawless performance through the entire operational duration of the test, while higher scores indicate worse performance. There is no upper bound on test scores. Though it might seem that zero would be the lower bound, in practice no athlete can achieve a perfect score.

Focus test scores from 849 athletes of ages 12-18 years with 46.4% female and 53.6% male were collected in spring 2015. Descriptive statistics are given in Table 1 for these 849 scores. All statistics were done using IBM SPSS Statistics version 22.0 (IBM Corp, Armonk, New York, United States). 

**Table 1:** Descriptives of focus normative data

			Statistic	Standard Error
SCORE	Mean		29633.050935	316.0808537
	95% Confidence Interval for Mean	Lower Bound	29012.658371	
		Upper Bound	30253.443499	
	Five percent trimmed mean		29430.565087	
	Median		28373.104796	
	Variance		84821133.041	
	Std. Deviation		9209.8389259	
	Minimum		11122.7562	
	Maximum		49960.5790	
	Range		38837.8229	
	Interquartile Range		13263.3268	
	Skewness		.396	.084
	Kurtosis		-.669	.168

The raw Focus score data were transformed based on z-score computation, with a mean = 50 and a standard deviation = 10. Computed T-scores were compared to immediate post-concussion assessment and cognitive testing (ImPACT) normative scores [6] and classification ranges were established from "mildly impaired" to "very superior" (see Table 2), with the majority of respondents falling into the "average" range of T-score equal to 44 to 56.99 (original n=366). Additionally, the Focus software was modified to shift these category parameters automatically with each addition to the baseline population.

**Table 2:** Thresholds for scoring focus derived from normative data

TSCORE AND ESTIMATED EYEGUIDE SCORE									
Classification Range	SCORE								
	Estimated T-score Range	Estimated Lower Bound	Estimated Upper Bound	Mean of Current Baseline Scores	Minimum Current Baseline	Maximum Current Baseline	Range Current Baseline	Standard Deviation Current Baseline	Standard Error Current Baseline
Very superior	< 29	0.00	11277.7	11163.22	11122.76	11203.68	80.92	57.22	40.46
Superior	30-36	11277.8	17702.1	15449.88	11413.60	17692.93	6279.33	1726.81	226.74
High average	37-43	17702.2	24126.4	21066.64	17711.17	24087.90	6376.73	1858.87	130.15
Average	44-56	24126.5	36057.4	29594.43	24177.71	36010.11	11832.40	3362.31	175.75
Low average	57-63	36057.5	42481.8	38980.08	36089.07	42236.53	6147.47	1825.23	184.38
Borderline	64-69	42481.9	47988.4	45239.10	42495.76	47904.03	5408.27	1582.91	180.39
Mildly impaired	70-75	47988.5	53495.0	49003.77	48181.30	49960.58	1779.28	552.51	108.36
Moderately impaired	76-79	53495.1	57166.0	N/A	N/A	N/A	N/A	N/A	N/A
Severely impaired	> 80	57166.1	None	N/A	N/A	N/A	N/A	N/A	N/A

**Figure 1:**
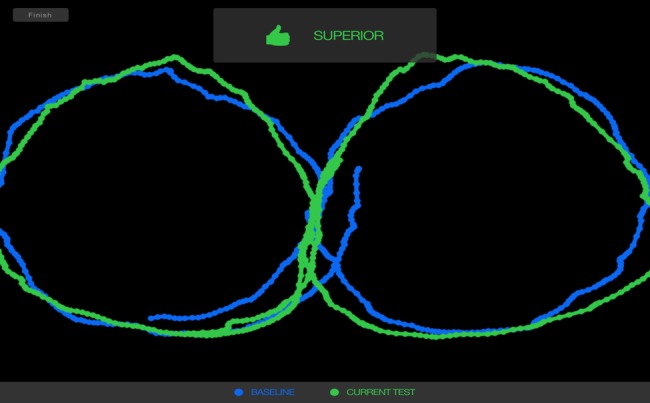
Focus test results showing superior score

**Figure 2:**
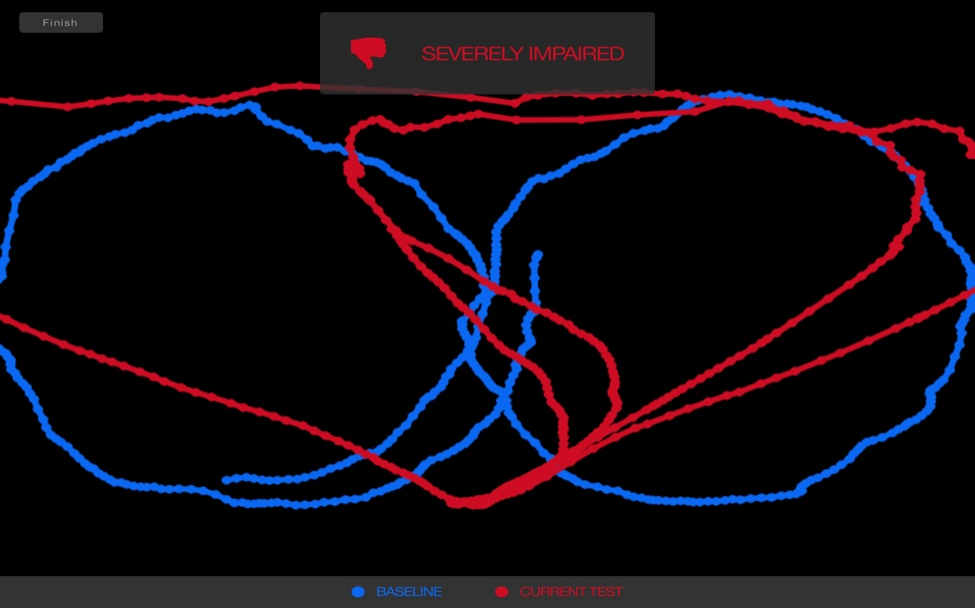
Focus test result shows impaired score

As part of a typical testing process, an athlete using Focus establishes an individual baseline. Then subsequent tests, such as those potentially occurring after injury are compared to that individual baseline. If the score is two category threshold ranges or standard deviations below the baseline, the Focus indicates the possibility of impairment (see Figures 1-2).

Because Focus is cloud-based, all new, uploaded Focus scores from athletes are used to update the threshold parameters so that they are current and reflective of the entire community of the tested athletes.

With more data, it is hoped that an "impaired" score in Focus can be correlated directly with a concussion. However, at present Focus is not designated as a medical device and cannot be used to detect concussion. But still, a feature in its software allows for test administrators to mark an impaired score with a “C” label for the concussion. This labeling is only done after a physician diagnoses concussion and the purpose is to establish an injury snapshot in Focus so that future testing after injury can be used along with other tools available to the test administrator to manage the return of the athlete to normal and thus dectect the eligibility for participation again in sports.

## Discussion

Now deployed at multiple locations across the United States, Focus, to date, has recorded more than 4,000 test scores. Of these, 2,736 have been designated as individual baseline scores. Two hundred and twenty-seven have been labeled as impaired. Ninty eight have been labeled with a “C”, indicating as noted before, that a physician has diagnosed the athlete in question with a concussion and the test administrator has marked an impaired score with “C” to signal a recovery monitoring start point.

**Figure 3:**
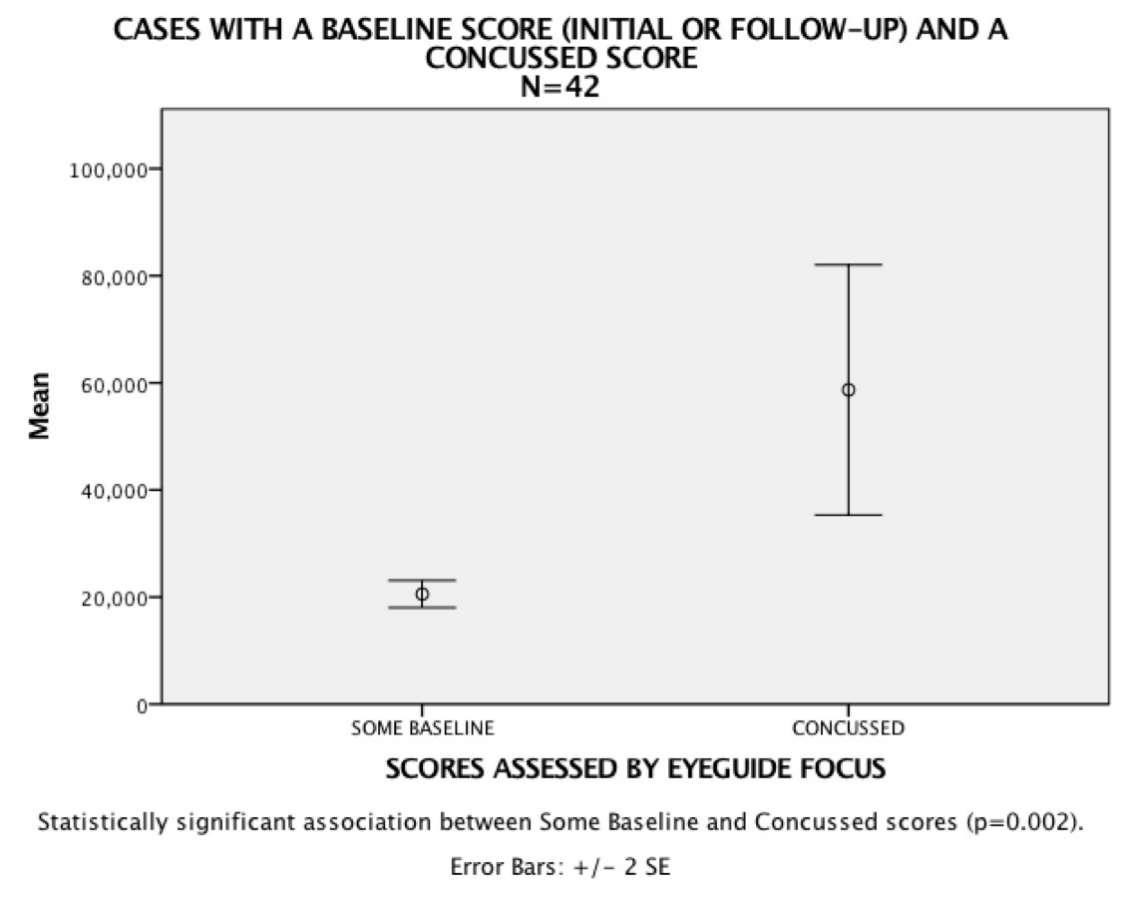
Test cases with baseline and concussion scores

Specifically, there are 42 instances of athletes recording an individual baseline test followed then by a severely impaired score that was later labeled as a concussed score. The analysis shows a statistical significance association between the baseline and concussed scores (see Figure 3).

**Figure 4:**
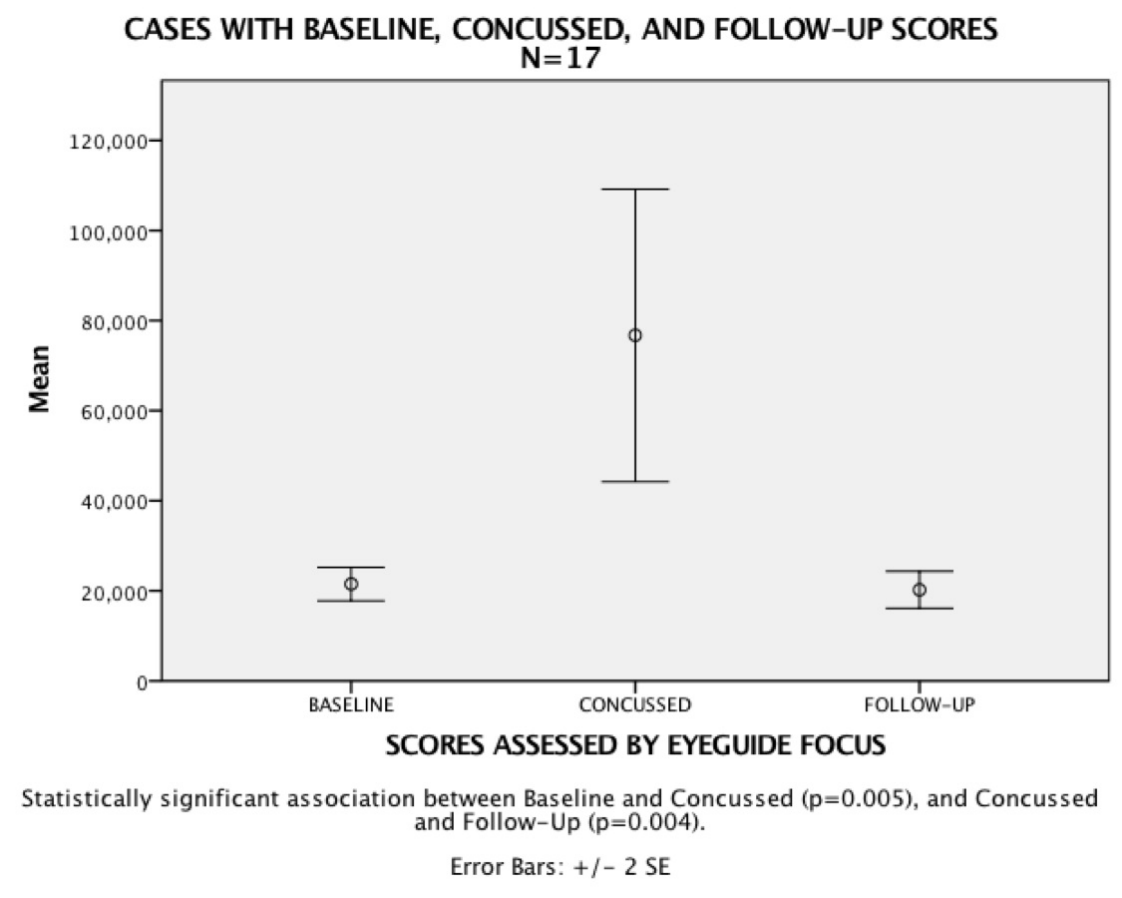
Test cases with baseline, concussion and follow-up normal scores

Further, 17 athletes with individual and impaired concussed scores also had follow-up test scores indicating a return to the same threshold within an average of two weeks, as the prior, pre-injury baseline score demonstrating that Focus has the potential to track healthy return to normal in athletes (see Figure 4).

**Figure 5:**
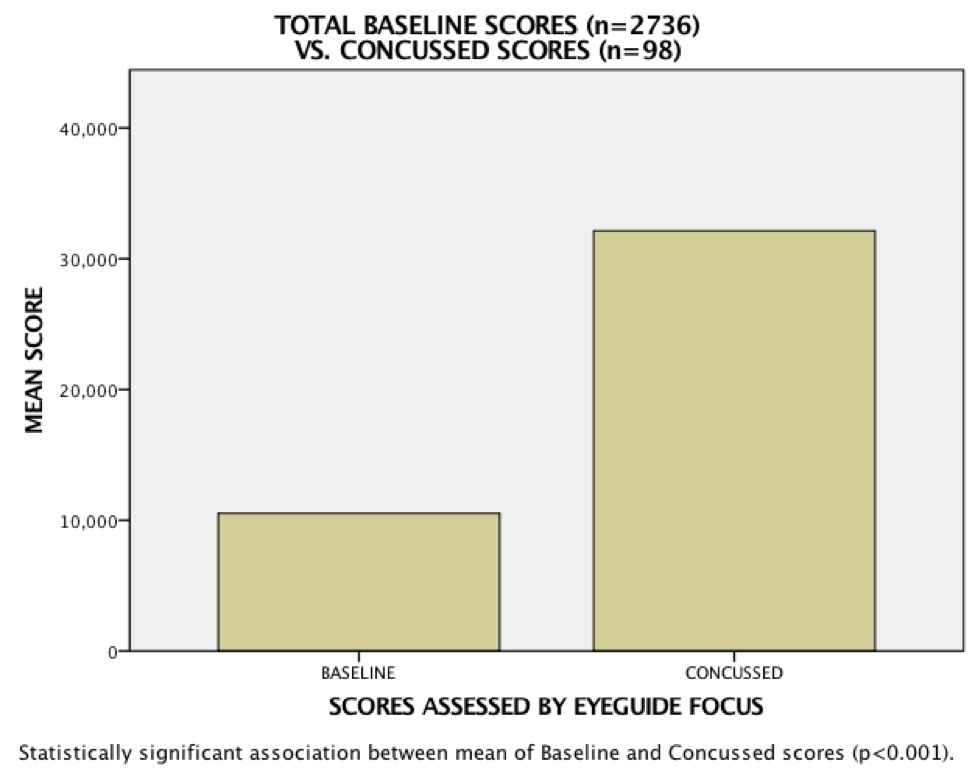
Analysis showing statistically significant difference between baseline and concussion scores

It is important to note that these results are not part of a controlled study. Test administrators were not tasked with employing a specific protocol for testing athletes before or after injury. There was no accounting for prescription drug use, lack of sleep, previous mTBI incidence, or other factors that might influence test scoring. In addition, although test administrators were trained in the use of Focus, there were no controls in place to require them to throw out test scores that may have been influenced by the poor test set-up, such as improper hardware use or distraction during testing. Nevertheless, a comparison of all baseline scores (n=2736) to all concussion scores (n=98) found a T-test P-value < 0.001, indicating a significant difference in means between the two (Figure 5).

## Conclusions

We performed a preliminary statistical analysis on data from a few thousand subjects to gauge the efficacy of EyeGuide Focus, a low-cost, ocular-based impairment test for assessing concussion. A comparison of all baseline scores (n=2736) to all concussion scores (n=98) found a T-test P-value < 0.001, indicating a significant difference in means between the two.

Additionally, there were 42 instances of athletes recording an individual baseline test followed by a severely impaired score that was later labeled as a concussed score. Analysis showed a statistical significance association between the baseline and concussed scores. Further, 17 athletes with individual and impaired concussed scores also had follow-up test scores indicating a return to the same threshold as the prior, pre-injury baseline score, demonstrating that Focus has the potential to track healthy return to normal in athletes.

Currently, independent researchers are managing more controlled studies to evaluate the efficacy of Focus among particular populations and sports.
